# Development of an Assessment Method for Investigating the Impact of Climate and Urban Parameters in Confirmed Cases of COVID-19: A New Challenge in Sustainable Development

**DOI:** 10.3390/ijerph17082801

**Published:** 2020-04-18

**Authors:** Behrouz Pirouz, Sina Shaffiee Haghshenas, Behzad Pirouz, Sami Shaffiee Haghshenas, Patrizia Piro

**Affiliations:** 1 Department of Mechanical, Energy and Management Engineering, University of Calabria, 87036 Rende, Italy; behrouz.pirouz@unical.it; 2 Department of Civil Engineering, University of Calabria, 87036 Rende, Italy; S.shaffiee@yahoo.com (S.S.H.); Sami.shaffiee@gmail.com (S.S.H.); patrizia.piro@unical.it (P.P.); 3 Department of Computer Engineering, Modelling, Electronics and Systems Engineering, University of Calabria, 87036 Rende, Italy

**Keywords:** sustainable development, climate and urban parameters, COVID-19, MLR

## Abstract

Sustainable development has been a controversial global topic, and as a complex concept in recent years, it plays a key role in creating a favorable future for societies. Meanwhile, there are several problems in the process of implementing this approach, like epidemic diseases. Hence, in this study, the impact of climate and urban factors on confirmed cases of COVID-19 (a new type of coronavirus) with the trend and multivariate linear regression (MLR) has been investigated to propose a more accurate prediction model. For this propose, some important climate parameters, including daily average temperature, relative humidity, and wind speed, in addition to urban parameters such as population density, were considered, and their impacts on confirmed cases of COVID-19 were analyzed. The analysis was performed for three case studies in Italy, and the application of the proposed method has been investigated. The impacts of parameters have been considered with a delay time from one to nine days to find out the most suitable combination. The result of the analysis demonstrates the effectiveness of the proposed model and the impact of climate parameters on the trend of confirmed cases. The research hypothesis approved by the MLR model and the present assessment method could be applied by considering several variables that exhibit the exact delay of them to new confirmed cases of COVID-19.

## 1. Introduction

Sustainable development is a concept which seeks to meet the needs of today’s generation without creating problems for the next generations. The sustainable development process consists of three main aspects, namely the environment, economy, and society, with the government policies to be considered as the fourth important aspect of this process from the current decade onwards [1,2]. Although the sustainable development process has a specific framework, there are many serious problems in implementing its process, and epidemic diseases are examples of this kind of problem, which can lead to temporary or permanent barriers and could affect the previous attempts on different criteria of sustainable development [3,4].

The coronavirus disease is one of the epidemic diseases that many governments have experienced in recent years. Hence, there are several studies of coronavirus disease in recent years [5,6,7,8,9,10]. Kampf et al. (2020) investigated the number of days of persistence of coronaviruses on inanimate surfaces. They found out that this virus can survive up for to 9 days [11]. Chinazzi et al. (2020) investigated the impact of travel restrictions on the spread of COVID-19. Their results show that travel restrictions can be very useful in decreasing the transmission of disease in the community [12]. Nkengasong and Mankoula (2020) evaluated the looming threat of COVID-19 from China to Africa. They investigated ways of transmitting the disease like trade between China and Africa, and also considered health-care systems. The obtained results show that for preventing a major disaster, Africa needs to be supported collectively, and fast [13].

Pandey et al. (2020) investigated the confirmed cases in India using two mathematical approaches, including the SEIR and regression models. Their models show a suitable performance in the predicate of the number of confirmed cases in short-term and long-term intervals, which would be useful for planning the health system in India [14]. Nazari Harmooshi (2020) investigated environmental conditions in the spread of COVID-19 and found that humidity and temperature play critical roles [15]. Pirouz and Violini (2020) assessed the outbreak patterns of COVID-19 by the investigation of 117 countries. Their results determine two mechanisms of normal and accumulation in the development of Coronavirus [16].

Gilbert et al. (2020) carried out a modeling study about the readiness and vulnerability of African countries against risks of COVID-19. Based on their results, they made some recommendations for some African countries with moderate to high risk of importation of COVID-19 [17]. Wen et al. (2020) investigated the role of the media and their effects on during times of crisis of the COVID-19 outbreak. Their results show that the biased and misleading coverage of mass media can constrain the positive efforts of treatment groups and of other groups involved in facing this crisis [18]. Hu et al. (2020) used artificial intelligence to predict the transmission period of COVID-19 in China. According to their results, artificial intelligence provided high-performance capacity in predicting the outbreak period [19]. In another study, Chen et al. (2020) proposed a time-dependent susceptible infected–recovered (SIR) model that is applicable for the prediction of the total number of confirmed cases [20]. Li and Feng investigated the trend of the COVID-19 outbreak in China. Their results show that quick and active strategies could be very effective in reducing and control the epidemic of COVID-19 [21].

Pirouz et al. (2020) evaluated the relationship between environmental parameters and confirmed cases of COVID-19, which is a serious challenge in the sustainable development process. They used an artificial intelligence algorithm to investigate several case studies. They found that there is a meaningful relationship between confirmed COVID-19 cases and environmental factors (urban and climate parameters) [22].

By reviewing the previous studies, it can be concluded that COVID-19 as an epidemic disease can create many barriers to economic, environmental, and social development, which in the involved countries might lead to a temporary or a long-term negative impact on sustainable development. The main aim of this study is to investigate the impact of climate factors on the confirmed COVID-19 cases and to propose a multivariate linear regression (MLR) model to improve the prediction.

## 2. Materials and Methods

We used two approaches to find out the possible correlations between the trends of confirmed cases, climate data, and previous positive cases, and according to the results, the final multivariate equations have been provided. The two approaches are as follows:

- The multivariate linear regression analysis in three regions in Italy with the highest confirmed cases of COVID-19;
- The trend analysis of the confirmed cases and climate parameters;

Conditions of analysis:

- The environmental and climate parameters in the analysis include density, average temperature, relative humidity, and wind speed;
- To decrease the impact of the different start dates of lockdown, different population density, and other unforeseen parameters in each region on the correlations between the confirmed cases and climate factors, a separate MLR analysis was done in each region.
- The climate data are based on the weather stations in the center of each region;
- The weather data of the three regions are presented in Table A1, Table A2 and Table A3 (See Appendix A);
- The analysis period is from 14 February 2020 to 14 March 2020 (1 month).

It must be mentioned that there is always some delay between the actual date when the case is infected and the registration as confirmed contagion in the media. The reasons are as follows:

- The estimated incubation period of COVID-19 is about 2–14 days [23]. However, the mean observed incubation periods were 3.0 days (study based on 1324 cases) and 5.2 days (based on 425 cases), [24,25];
- According to the policy for the COVID-19, not everybody will be tested, especially not those with no symptoms [26]. The NHS, UK is only testing people with symptoms such as a fever, cough, or shortness of breath, [27];
- The results of the laboratory are typically available within one day (24 h) [28,29]. In Lombardy, there are some delays in testing, and the data may not reflect the actual numbers [30]. Moreover, the daily information of the confirmed cases refers to the previous 24 h, [31].

### 2.1. Case Study

**Figure 1:**
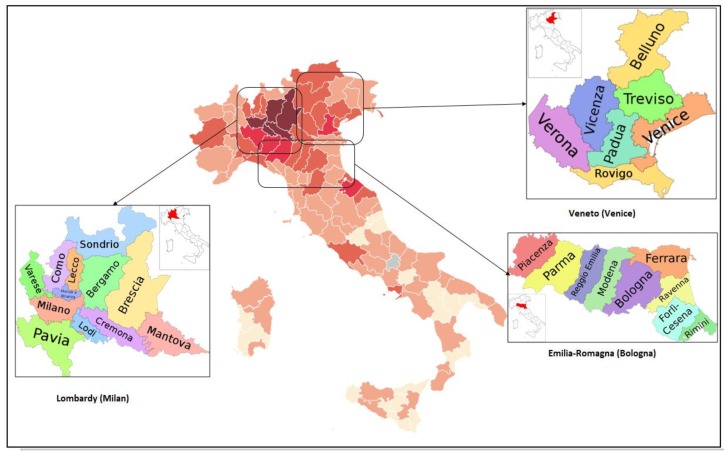
The case studies location, Italy [37,38,39,40].

To carry out the analysis, the dataset of three regions in Italy, namely Lombardy (Milan), Veneto (Venice), and Emilia-Romagna (Bologna), are displayed in Table 1, and the location of the case studies is shown in Figure 1.

The lockdown could affect the trend of positive cases, and after around 13–14 days from full lock down the number of confirmed cases is expected to decrease, as mentioned in a previous study by [22]. The dates of quarantine in Italy can be seen in Table 2.

### 2.2. Mathematical Modeling and Multivariate Linear Regression Analysis

Mathematical modeling has been used successfully for modeling many natural and human-made hazards [44,45,46,47,48]. One of the most important aspects of any optimization is the expression of the problem in the form of a mathematical model. Due to the complexity of the problems, different approaches may be used to express a problem as a mathematical model [49,50,51,52]. Many studies have been carried out in various fields of science to obtain mathematical models and solutions for determining problems [53,54,55]. To be able to develop a prediction model, one method can be regression analysis. By regression analysis, the effect of two or more variables on the dependent variable can be assessed. Multivariate linear regression (MLR) is one of the most applicable mathematical methods to determine a linear relationship between independent and dependent parameters [56,57]. The MLR model for the ith sample is as Equation (1):

y_i_ = β_0_ + β_1_x_i__1_ + β_2_x_i__2_ +…+ β_j_x_in_ + ε_i_ (1)

where:

ε is random error βi, (i = 0, 1, …, n) are the regression coefficients

In the equation, the dependent variable, y, linearly depends on a combination of independent variables, x.

In the analysis, the linearity, invariability, and normality of datasets are essential, along with the independence of observations. The final correlation can be evaluated through R^2^ and the regression beta coefficient. The beta coefficients are the degree of change by each variable in the outcome and can be positive or negative. The highest beta coefficient means the maximum possible impact and can be used in a regression model to compare the relative importance of each coefficient. [56,57].

### 2.3. The SPSS Model Set Up

To conduct advanced analyses, SPSS (IBM, Armonk, NY, USA) has been used. The critical factors in the SPSS Model set up are as follows:

- The main factor that affects the future positive cases would be the positive cases of previous days up to 14 days before, since the incubation period of COVID-19 is about 2–14 days.
- The second factor in the positive cases is the weather condition, and this might be the reason why the graphs of confirmed cases exhibit short-time fluctuations.
- Shifting of variables to confirmed cases from one to nine days, in accordance with the existing delay between the actual date of infection and the announcement of confirmed cases, as mentioned above in the conditions of analysis.

The independent and dependent variables in the model are as follows:

- x1: Average Temperature [°C]
- x2: Humidity [%]
- x3: Wind [km/h]
- x4: Total number of the confirmed cases in 14 days
- Y: Daily confirmed cases (dependent variable)

## 3. Results

This section is divided by subheadings. It will provide a concise and precise description of the experimental results, their interpretation, and the experimental conclusions that can be drawn.

### 3.1. The Multivariate Linear Regression Analysis

The results of multivariate linear regression for three case studies can be seen in Table 3, Table 4 and Table 5. The tables show the regression results, including *p*-value and beta coefficients for the variables x1, x2, x3, and x4, as well as R^2^ and the *p*-value of the overall regression models by shifting the variables with respect to confirmed cases of COVID-19 from one to nine days. The results show that the effect of the independent variable x4 was significant in all models. Although the effect of the variables x1, x2, x3 in some intervals was not significant, the R^2^ values and the significance level of the regression models indicate the confirmation of these regression models.

As it is clear from Table 3, Table 4 and Table 5, the accuracy of the model in the three case studies depends on the specific delays between independent and dependent variables.

To form a regression model that includes the maximum effect of independent variables x1, x2, x3 on the dependent variable, the time intervals with the highest beta coefficient (maximum possible effect) for each of three variables are specified and presented in Table 6.

### 3.2. The Correlations between the Confirmed Cases and Climate Factors

**Figure 2:**
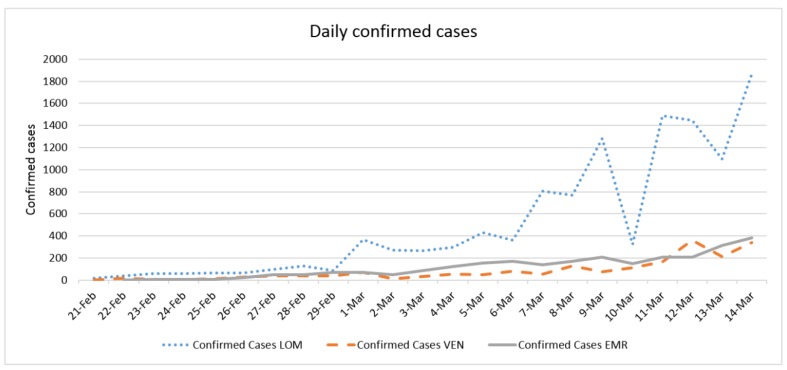
The daily confirmed cases of COVID-19 in three case studies.

**Figure 3:**
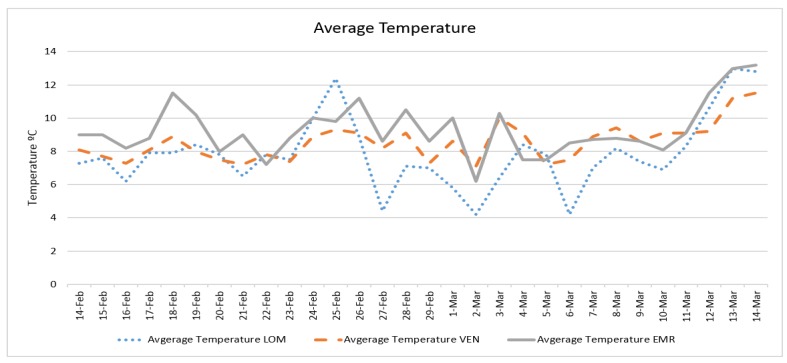
The daily average temperature in three case studies.

**Figure 4:**
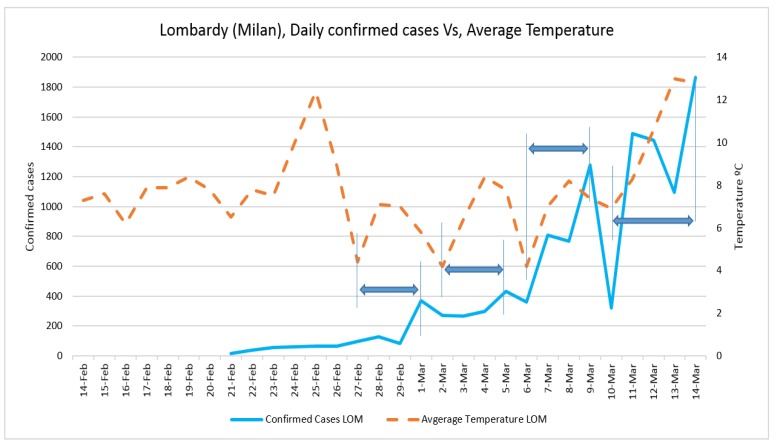
Daily confirmed cases of COVID-19 in Lombardy and average temperature.

**Figure 5:**
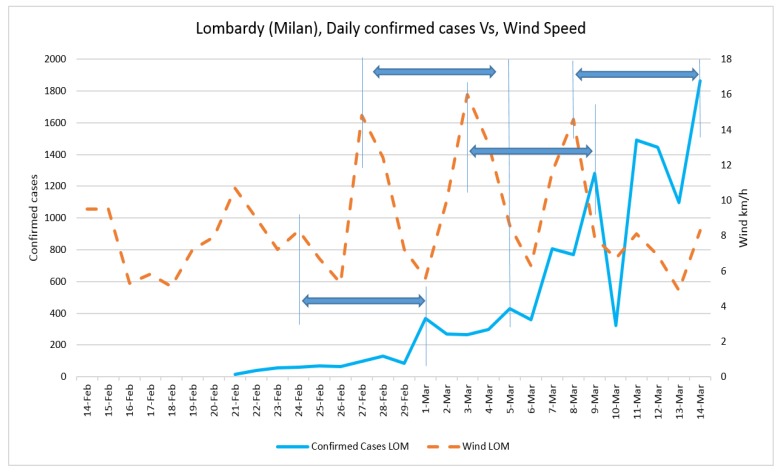
Daily confirmed cases of COVID-19 in Lombardy and wind speed.

Daily confirmed cases and the average temperature in three case studies are presented in Figure 2 and Figure 3, respectively. The trend of daily positive cases of COVID-19 in Lombardy and average temperature and wind speed are shown in Figure 4 and Figure 5. The blue arrows in each graph are at the same length (a specific period, for example, four days), which means a repeat of impact happened. However, since the impact of wind, humidity, and temperature are all together, in some rare cases, the higher impact of one (a severe wind) could be dominant in comparison with other variables.

**Figure 6:**
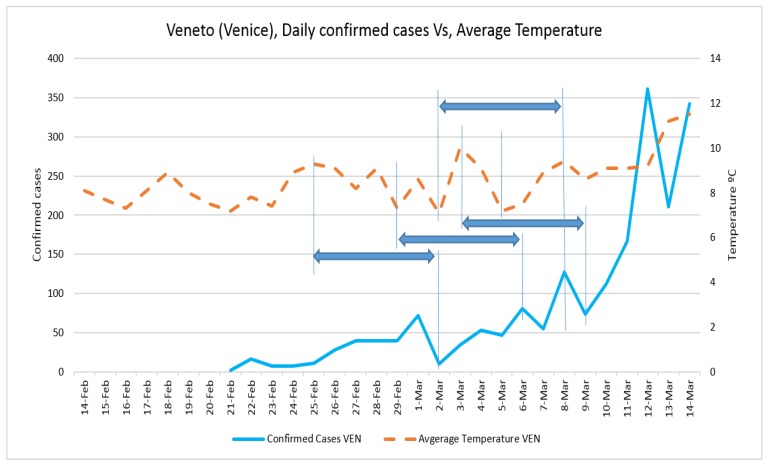
Daily confirmed cases of COVID-19 in Veneto and average temperature.

**Figure 7:**
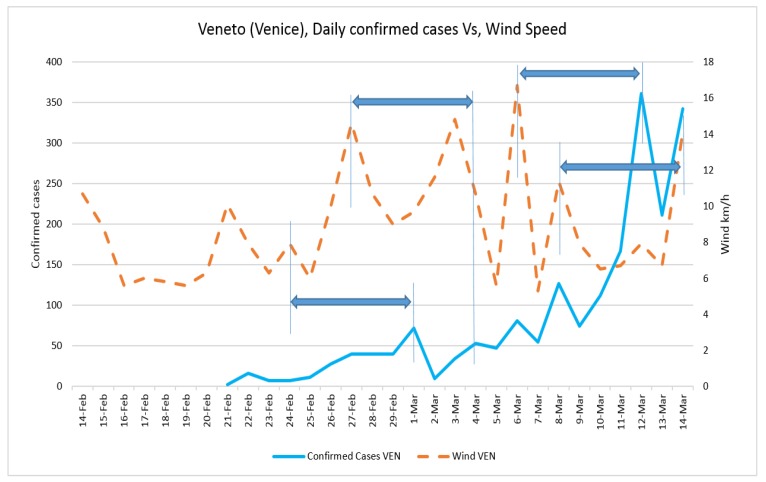
Daily confirmed cases of COVID-19 in Veneto and wind speed.

**Figure 8:**
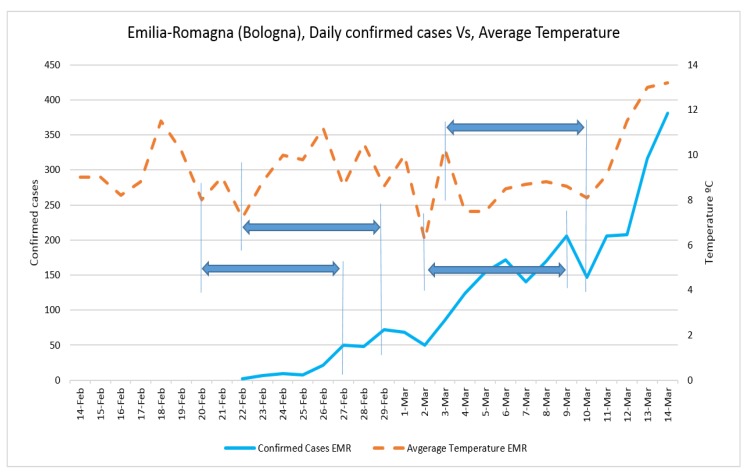
Daily confirmed cases of COVID-19 in Emilia-Romagna and average temperature.

**Figure 9:**
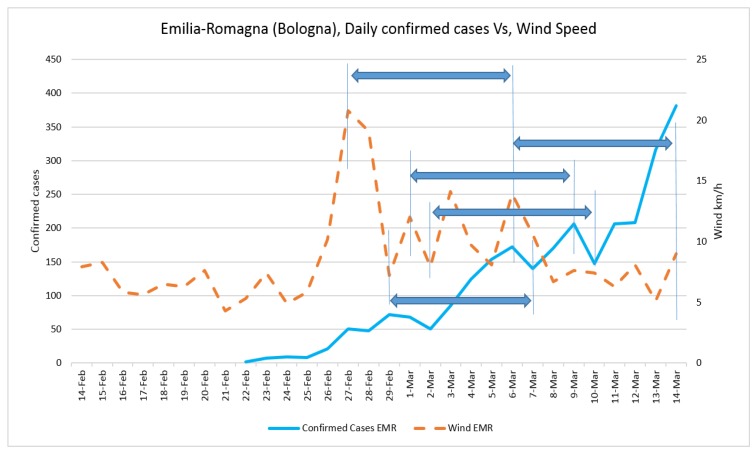
Daily confirmed cases of COVID-19 in Emilia-Romagna and wind speed.

The fluctuation of daily positive cases of COVID-19 in Veneto and Emilia-Romagna in comparison with average temperature and wind speed are shown in Figure 6, Figure 7, Figure 8 and Figure 9.

### 3.3. The Final Multivariate Equations

According to the best configuration of the model, the final multivariate equations have been provided, and the coefficients, significance level of constant, independent variables, and standardized coefficients beta are displayed in Table 7.

The values of R^2^ in all of these regression models are an appropriate level, and these regression models are significant (*p*-value = 0.000). In the case of variables x1, x2, x3 (with the high R^2^ and very desirable and meaningful regression models), and due to the main goal of the study, more emphasis is placed on beta coefficients. However, the results show a significant effect of variable x2 in Milan and also a significant effect of variable x3 in Venice. The final multivariate equations for each of the three case studies are presented in Table 8.

## 4. Discussion

The results of initial multivariate linear regression for three case studies show high accuracy. However, the accuracy changed by shifting the variables from one to nine days prior to observations. The shifting of variables with respect to confirmed cases reflected the fact that the positive cases are defined as positive-test cases with symptoms such as fever, cough, or shortness of breath, and the symptoms of COVID-19 occur about 3 to 5 days earlier. Moreover, there are two other reasons for delays, each of about one day, namely the delay in knowing the results of the laboratory analysis and the 24 hour delay between the confirmation of the infection and its announcement. After consideration of the impacts during nine days, the best combination was determined, and the final MLR model was developed accordingly. Comparisons between the final multivariate equations we obtained and previous ones by R^2^, and the coefficients and significance level of the constant show that accuracy was improved by the proposed method. The hypothesis of the study approved by the MLR model and the assessment method could be used by consideration of any new variables to exhibit the exact delay of each of them to new confirmed cases of coronavirus.

The trend analysis showed that the number of confirmed cases in all three case studies was equal on 29 February; however, in Lombardy, it grew more in the following days. This might be due to the population density of Lombardy (422) being larger those of Veneto (272) and Emilia-Romagna (199) and to the fact that the average temperature of Lombardy was lower than in the other two regions since 25 February.

The number of confirmed cases in Emilia-Romagna was equal to that of Veneto until 2 March, increasing nearly with the same trend, and this might be because the average temperatures of the two regions were similar before 2 March. The fluctuation of daily positive cases of COVID-19 in comparison with average temperature and wind speed in all three case studies also showed the correlations with some delay and proved the results of regression analysis.

It must be mentioned that the equations we provided are a regression based on the multivariable technique. They present the best combination of all factors in prediction, not each of them separately. Therefore, the impact of one can overcome the other. For example, a wind with very high speed might be dominant with respect to a small decrease in average temperature. In addition, the developed prediction equations are based on the dataset of three case studies that were in an increasing trend of positive cases of COVID-19. Therefore, they cannot be used to predict the behavior of positive case in a future decreasing trend. However, the same delays for the impact of climate factors would also exist in a decreasing trend. As mentioned in [22], due to the incubation period of COVID-19, which is about 2–14 days, it should be expected that the increasing trend would stop in a period, which, according to Chinese experience, would be expected to be about 13 days from the starting date of full lockdown. However, in the Italian case, it will probably last longer because of the different features of the lockdown implementation. Finally, to improve the accuracy of the prediction, the same analysis with the mentioned condition and by consideration of delays are suggested for future studies using MnLR and artificial intelligence techniques.

## 5. Conclusions

In this study, an assessment method for investigating the impact of climate and urban parameters in confirmed cases of COVID-19, as a new challenge in sustainable development was investigated using the multivariate linear regression and trend analysis. The results of MLP for three case studies in Italy showed a high accuracy correlation between the variables and observations. However, the accuracy changed by shifting the impact time. The results of the analysis demonstrate the effectiveness of the proposed model and the impact of climate parameters on the trend of confirmed cases. The results of shifting the variables with respect to confirmed cases demonstrate that there is a delay from four to eight days between the impact of weather parameters and the new confirmed cases. The reason could be the fact that the positive cases are among those with symptoms, and the symptoms of COVID-19 occur after about 3 to 5 days, and there are also two other delays, each of about one day, including the results of the laboratory analysis and the announced time that belongs to the previous 24 h. Comparisons between the final developed MLR and the first ones showed an improvement in the accuracy of the proposed technique. According to the trend analysis, it seems that the population density and weather conditions could affect the daily positive cases of COVID-19. The fluctuations of daily positive cases in all three case studies confirmed the impact of climate factors with some delay and proved the results of MLR. As a result, the developed prediction model can be applied as an assessment method for investigating the impact of climate and urban parameters in confirmed cases of COVID-19.

## Figures and Tables

**Table 1:** The selected case studies.

Case Study	Population [32,33]	Density, Population/km^2^ [34,35]	Total Confirmed Cases Until 15th March [36,37]
Lombardy (Milan)	10,060,574	422	13,272
Veneto (Venice)	4,905,854	272	2172
Emilia-Romagna (Bologna)	4,459,477	199	3093

**Table 2:** The lockdown program in Italy to the spread of Coronavirus, [41,42,43].

Region	Start Date
Eleven municipalities in Lombardy and Veneto	22 February
All of Lombardy and 14 other northern provinces.	8 March
Entire Italy (travel restrictions and public gatherings)	10 March
Entire Italy (all commercial activity except pharmacies and supermarkets)	11 March

**Table 3:** The result of multivariate linear regression, Lombardy (Milan).

Independent Variables		Shifted Variables to Confirmed Cases [Days]								
		9	8	7	6	5	4	3	2	1
x1: Average Temperature [°C]	*p*-value	0.457	0.918	0.628	0.805	0.594	0.358	0.486	0.451	0.618
	Beta	−0.105	0.020	−0.070	−0.031	−0.073	0.115	−0.083	−0.084	−0.063
x2: Humidity [%]	*p*-value	0.549	0.873	0.126	0.086	0.492	0.978	0.586	0.734	0.847
	Beta	−0.115	−0.032	−0.250	0.264	0.106	−0.004	−0.072	0.041	−0.025
x3: Wind [km/h]	*p*-value	0.778	0.930	0.185	0.563	0.797	0.215	0.993	0.464	0.954
	Beta	0.061	0.018	−0.219	0.089	0.040	0.176	−0.001	−0.092	0.008
x4: Total of confirmed case in 14 days	*p*-value	0.002	0.001	0.000	0.000	0.000	0.000	0.000	0.000	0.000
	Beta	0.791	0.869	0.939	0.866	0.877	0.897	0.894	0.914	0.924
R^2^		0.750	0.734	0.823	0.840	0.801	0.826	0.828	0.843	0.806
*p* value of Model (ANOVA test)		0.008	0.006	0.000	0.000	0.000	0.000	0.000	0.000	0.000

**Table 4:** The result of multivariate linear regression, Veneto (Venice).

Independent Variables		Shifted Variables to Confirmed Cases [Days]								
		9	8	7	6	5	4	3	2	1
x1: Average Temperature [°C]	*p*-value	0.530	0.813	0.597	0.113	0.384	0.220	0.775	0.493	0.634
	Beta	0.115	0.040	−0.056	−0.214	0.111	0.157	0.036	0.082	0.061
x2: Humidity [%]	*p*-value	0.555	0.740	0.359	0.785	0.809	0.905	0.787	0.795	0.998
	Beta	0.100	−0.055	−0.100	0.035	0.030	−0.015	−0.032	−0.029	0.000
x3: Wind [km/h]	*p*-value	0.789	0.809	0.004	0.285	0.053	0.335	0.216	0.692	0.154
	Beta	0.049	0.045	−0.375	0.141	−0.259	−0.118	−0.152	−0.045	−0.161
x4: Total of confirmed case in 14 days	*p*-value	0.001	0.001	0.000	0.000	0.000	0.000	0.000	0.000	0.000
	Beta	0.838	0.849	0.945	0.881	0.874	0.837	0.850	0.861	0.818
R^2^		0.766	0.755	0.887	0.823	0.813	0.808	0.799	0.809	0.816
*p* value of Model (ANOVA test)		0.006	0.004	0.000	0.000	0.000	0.000	0.000	0.000	0.000

**Table 5:** The result of multivariate linear regression, Emilia-Romagna (Bologna).

Independent Variables		Shifted Variables to Confirmed Cases [Days]								
		9	8	7	6	5	4	3	2	1
x1: Average Temperature [°C]	*p*-value	0.270	0.120	0.861	0.454	0.684	0.827	0.868	0.336	0.200
	Beta	0.172	0.178	-0.019	0.075	0.042	−0.024	0.017	0.080	0.107
x2: Humidity [%]	*p*-value	0.328	0.212	0.784	0.072	0.226	0.600	0.433	0.139	0.320
	Beta	−0.175	−0.164	−0.037	−0.228	−0.144	−0.062	0.087	0.140	0.088
x3: Wind [km/h]	*p*-value	0.316	0.880	0.182	0.798	0.667	0.488	0.479	0.057	0.227
	Beta	−0.183	0.019	0.186	−0.029	−0.049	−0.082	0.080	0.186	0.112
x4: Total of confirmed case in 14 days	*p*-value	0.000	0.000	0.000	0.000	0.000	0.000	0.000	0.000	0.000
	Beta	1.028	1.037	0.906	1.018	0.967	0.923	0.942	0.967	0.938
R^2^		0.870	0.925	0.905	0.909	0.889	0.872	0.874	0.908	0.909
*p* value of Model (ANOVA test)		0.001	0.000	0.000	0.000	0.000	0.000	0.000	0.000	0.000

**Table 6:** The best configuration of the model for the equation.

Case Study	Temperature	Humidity	Wind	Total 14 Days of Positive Cases
Lombardy (Milan)	−4 days	−6 days	−7 days	From −2 days
Veneto (Venice)	−6 days	−7 days	−7 days	From −4 days
Emilia-Romagna (Bologna)	−8 days	−6 days	−7 days	From −8 days

**Table 7:** The final coefficients, beta and significance of constant for independent variables.

Case Studies	Variables	Coefficients	Beta	* p * Value
Lombardy (Milan)	Constant	−594.318	----	0.128
	x1	10.129	0.034	0.727
	x2	7.091	0.255	0.031
	x3	17.592	0.102	0.383
	x4	0.204	0.904	0.000
	R^2^	0.877		
	*p* value of Model (ANOVA test)	0.000		
Veneto (Venice)	Constant	241.580	----	0.006
	x1	−17.168	−0.153	0.121
	x2	−0.299	−0.034	0.711
	x3	−10.442	−0.349	0.001
	x4	0.422	1.041	0.000
	R^2^	0.922		
	*p*-value of Model (ANOVA test)	0.000		
Emilia-Romagna (Bologna)	Constant	−2.009	----	0.983
	x1	10.089	0.154	0.130
	x2	−0.508	−0.109	0.376
	x3	3.054	0.161	0.139
	x4	0.330	1.019	0.000
	R^2^	0.935		
	*p*-value of Model (ANOVA test)	0.000		

**Table 8:** The provided equations in three case studies.

Case Study	Multivariate Equations
Lombardy (Milan)	$y=-594.318+10.129x_{1}+7.091x_{2}+7.091x_{3}+0.204x_{4}$
Veneto (Venice)	$y=241.580-17.168x_{1}-0.299x_{2}-10.442x_{3}+0.422x_{4}$
Emilia-Romagna (Bologna)	$y=-2.009+10.089x_{1}-0.508x_{2}+3.054x_{3}+0.330x_{4}$

## Appendix A

**Table A1:** The dataset of Lombardy (Milan), [58,59].

Date	Average Temperature [°C]	Humidity [%]	Wind [Km/h]	Confirmed Cases
14-Feb	7.3	70.1	9.5	-
15-Feb	7.6	61.4	9.5	-
16-Feb	6.2	80.7	5.3	-
17-Feb	7.9	81.1	5.8	-
18-Feb	7.9	79.4	5.1	-
19-Feb	8.4	66.1	7.2	-
20-Feb	7.8	43.9	7.9	-
21-Feb	6.5	46.3	10.7	15
22-Feb	7.8	64.6	9	40
23-Feb	7.5	69.3	7.2	57
24-Feb	10	75.8	8.3	61
25-Feb	12.4	79.2	6.7	67
26-Feb	8.9	81.1	5.3	65
27-Feb	4.4	47.6	14.8	98
28-Feb	7.1	43.2	12.4	128
29-Feb	7	37.6	7.2	84
1-Mar	5.8	94.4	5.6	369
2-Mar	4.2	96.3	10	270
3-Mar	6.4	82.1	16	266
4-Mar	8.4	36.1	13.2	300
5-Mar	7.8	63	8.6	431
6-Mar	4.2	96.9	6.3	361
7-Mar	7	65.1	11.6	808
8-Mar	8.2	37.7	14.6	769
9-Mar	7.4	61.9	7.9	1280
10-Mar	6.9	69	6.7	322
11-Mar	8.3	57.1	8.1	1489
12-Mar	10.6	67.6	6.9	1445
13-Mar	13	84.2	4.9	1095
14-Mar	12.8	83.6	8.3	1865

**Table A2:** The dataset of Veneto (Venice), [58,59].

Date	Avgerage Temperature [℃]	Humidity [%]	Wind [Km/h]	Confirmed Cases
14-Feb	8.1	89.4	10.7	-
15-Feb	7.7	83.3	8.8	-
16-Feb	7.3	82.4	5.6	-
17-Feb	8.1	92.2	6	-
18-Feb	8.9	89.9	5.8	-
19-Feb	8	86.7	5.6	-
20-Feb	7.5	74.3	6.3	-
21-Feb	7.2	71.3	10.1	2
22-Feb	7.8	68.2	7.9	16
23-Feb	7.4	87.6	6.3	7
24-Feb	8.9	86.4	7.9	7
25-Feb	9.3	92.7	6	11
26-Feb	9.1	90	10	28
27-Feb	8.2	62.6	14.6	40
28-Feb	9.1	52.5	10.7	40
29-Feb	7.3	64.2	9	40
1-Mar	8.6	79.8	9.7	72
2-Mar	7.1	94.8	11.6	10
3-Mar	10	89.8	14.8	34
4-Mar	9.1	76.2	10.7	53
5-Mar	7.2	72.8	5.6	47
6-Mar	7.5	80.7	16.7	81
7-Mar	8.9	82.5	5.3	55
8-Mar	9.4	68	11.4	127
9-Mar	8.6	68.2	7.9	74
10-Mar	9.1	74.2	6.5	112
11-Mar	9.1	82.3	6.7	167
12-Mar	9.2	84.9	7.9	361
13-Mar	11.2	89.8	6.7	211
14-Mar	11.5	73.8	14.1	342

**Table A3:** The dataset of Emilia-Romagna (Bologna), [58,59].

Date	Avgerage Temperature [℃]	Humidity [%]	Wind [Km/h]	Confirmed Cases
14-Feb	9	69.9	7.9	
15-Feb	9	57.9	8.3	
16-Feb	8.2	70.6	5.8	
17-Feb	8.8	77.5	5.6	
18-Feb	11.5	82.9	6.5	
19-Feb	10.2	83.3	6.3	
20-Feb	8.0	72.9	7.6	
21-Feb	9	74	4.3	
22-Feb	7.2	74.2	5.3	2
23-Feb	8.8	73.2	7.4	7
24-Feb	10	77.6	4.9	9
25-Feb	9.8	84.3	5.8	8
26-Feb	11.2	69.6	10.2	21
27-Feb	8.6	33.6	20.8	50
28-Feb	10.5	37.8	19.1	48
29-Feb	8.6	36.1	7.2	72
1-Mar	10	66.2	12	68
2-Mar	6.2	90.5	7.9	50
3-Mar	10.3	82.8	14.1	85
4-Mar	7.5	84.9	9.7	124
5-Mar	7.5	69	8.1	154
6-Mar	8.5	82.5	13.9	172
7-Mar	8.7	81.3	10.6	140
8-Mar	8.8	74.3	6.7	170
9-Mar	8.6	59.9	7.6	206
10-Mar	8.1	70.6	7.4	147
11-Mar	9.1	62.9	6.3	206
12-Mar	11.5	61.7	8.1	208
13-Mar	13	74	5.1	316
14-Mar	13.2	79.6	9	381
